# Mu-opioid and nociceptin receptors show divergent, cell-type-specific actions in the mesocorticolimbic reward system in opioid use disorder

**DOI:** 10.3389/fncel.2026.1774384

**Published:** 2026-04-10

**Authors:** Marie-Charlotte Allichon, Jeanne Espinosa, Rebecca H. Cole, Mei-Chuan Ko, Peter Vanhoutte, Max E. Joffe

**Affiliations:** 1 Department of Psychiatry, University of Pittsburgh, Pittsburgh, PA, United States; 2 Translational Neuroscience Program, University of Pittsburgh, Pittsburgh, PA, United States; 3 Sorbonne University, Centre National de la Recherche Scientifique, Institut National de la Santé et de la Recherche Médicale, Paris, France; 4 Institute of Biology Paris-Seine, Center for Neuroscience Sorbonne University, Paris, France

**Keywords:** addiction, GPCR, motivation, plasticity, stress regulation

## Abstract

The mu-opioid receptor (MOR) and the nociceptin/orphanin FQ receptor (NOPR) are closely related yet functionally distinct modulators of rewards, motivation, and affect. Within the mesocorticolimbic system, including the prefrontal cortex (PFC), the ventral tegmental area (VTA) and the nucleus accumbens (NAc), these receptors exhibit divergent, cell type–specific expression patterns that drive opposing behavioral outcomes. For example, MOR activation enhances rewards processing and reinforcement by facilitating dopamine transmission, whereas NOPR signaling in the VTA can reduce dopamine cell activity. In addition, MOR and NOPR are positioned within cortical circuits to preferentially reduce GABA and glutamate transmission, respectively. This review synthesizes current knowledge on how MOR and NOPR coordinate motivational and affective states through distinct neuronal populations across the mesocorticolimbic circuit. We also discuss emerging evidence for functional interactions between these systems and the therapeutic implications of pharmacological strategies targeting both receptors, including dual-acting MOR/NOPR ligands that enhance analgesic efficacy with reduced abuse liability. By integrating behavioral, molecular, and circuit-level findings, this synthesis aims to clarify how MOR and NOPR signaling jointly shape rewards and stress pathways, and provide insight into the development of safer and more effective treatments for opioid use disorders.

## Introduction

### The classical opioid receptor family

Chronic perturbation of the opioid system, whether by exogenous opiates (e.g., morphine, fentanyl) or dysregulation of endogenous neuropeptides [reviewed by Emery and Akil (2020), Higginbotham et al. (2022)], induces long-lasting adaptations that underlie the transition from recreational to maladaptive use (for a review see Koob and Volkow, 2010; Valentino and Volkow, 2018). To frame these neuroadaptations, it is useful to first outline the organization and function of the opioid receptor family.

The opioid system comprises four main G protein-coupled receptors (GPCRs): the mu-opioid receptor (MOR), delta-opioid receptor (DOR), kappa-opioid receptor (KOR), and the most recently identified nociceptin/orphanin FQ receptor (NOPR) (Kieffer and Evans, 2009; Toll et al., 2016). MOR, DOR, and KOR were pharmacologically characterized in the 1970s based on differential affinities of morphine-like compounds and endogenous opioid peptides such as endorphins, enkephalins, and dynorphins (Goldstein et al., 1979; Kieffer and Gavériaux-Ruff, 2002; Lord et al., 1977). Molecular cloning in the early 1990s confirmed that each receptor is encoded by a distinct gene (*Oprm1*, *Oprd1*, *Oprk1*), yet all share structural similarity and canonical G_i/o_-coupled signaling mechanisms including inhibition of adenylyl cyclase and activation of potassium channels (Charbogne et al., 2014; Chen et al., 1993; Evans et al., 1992; Kieffer et al., 1992; Kieffer and Gavériaux-Ruff, 2002; Law et al., 2000; Minami et al., 1993). Among these, MOR is the principal mediator of opioid analgesia and reinforcement. Activation of MOR by endogenous or exogenous opioids induces potent analgesia through inhibition of nociceptive transmission. In parallel, MOR activation enhances dopaminergic transmission in mesolimbic pathways, reinforcing rewards-related behaviors (Fields and Margolis, 2015; Le Merrer et al., 2009; Matthes et al., 1996). DOR and KOR, while sharing overlapping distributions, play distinct roles: DOR activation contributes to mood regulation and anxiolysis, whereas KOR signaling is classically associated with dysphoria and stress-induced aversion (Bruchas et al., 2010; Farahbakhsh and Siciliano, 2026; Pradhan et al., 2011; Van’t Veer and Carlezon, 2013). More recently, the orphan GPCR GPR139 has emerged as a non-canonical opioid receptor (Chan and Ogawa, 2025; Liu et al., 2015; Roy et al., 2024). Some authors have also proposed a Zeta receptor in early developmental studies (Zagon et al., 1991), though its functional relevance remains highly speculative. For a comprehensive review of the physiology and function of all opioid receptor systems in rodents and humans see (Mathis et al., 2025).

### Discovery of the nociceptin/orphanin FQ system

The discovery of the nociceptin/orphanin FQ (N/OFQ) system marked a turning point in opioid research. In 1994, Mollereau et al. (1994) cloned a GPCR of 370 amino acids that shares significant sequence homology with classical opioid receptors but does not respond to opioid peptides or naloxone, leading to its classification as an orphan receptor (Bunzow et al., 1994; Fukuda et al., 1994; Lachowicz et al., 1995; Nishi et al., 1994; Pan et al., 1995; Wang et al., 1994). In 1995, two groups independently identified the endogenous ligand of this receptor from brain extracts, giving rise to what is now known as N/OFQ and deorphanizing the receptor (Meunier et al., 1995; Reinscheid et al., 1995). Indeed, Meunier et al. (1995) isolated a 17–amino acid peptide (FGGFTGARKSARKLAMQ) from rat brain that produced a pronociceptive effect, rather than analgesia, when administered via intracerebroventricular injection and named it “nociceptin” to reflect this property. In parallel, Reinscheid et al. (1995) purified the same amino acid sequence from porcine brain extracts and called it “orphanin FQ,” referencing both the orphan receptor (ORL1) and the amino acids at its N- and C-termini (F/Q). Subsequent studies confirmed nociceptin and orphanin FQ to be identical and conserved across species, including bovine brain (Okuda-Ashitaka et al., 1996). The N/OFQ peptide, encoded by the prepronociceptin (*Pnoc)* gene, is structurally related to classical opioid peptides such as endorphins and enkephalins but does not activate MOR, DOR, or KOR (Anand et al., 2016; Mollereau et al., 1994; El Daibani and Che, 2022; Lambert, 2008; Reinscheid et al., 1995; Toll et al., 2016).

### NOPR is the fourth opioid receptor

Nociceptin/orphanin FQ receptor, encoded by *Oprl1*, is now considered the fourth member of the opioid receptor family (Calo et al., 2000). Although NOPR shares homology and overlapping distribution with MOR within rewards-related brain regions, NOPR modulates synaptic transmission in a distinct and sometimes opposite manner to MOR (Cippitelli et al., 2020; Kallupi et al., 2017; Koob and Volkow, 2016). Within the mesocorticolimbic circuit, including the prefrontal cortex (PFC), the ventral tegmental area (VTA) and the nucleus accumbens (NAc), MOR and NOPR show complementary expression patterns to regulate dopaminergic tone, synaptic plasticity, and the fine-tuning of rewards processing and stress responsivity (Driscoll et al., 2020; Fields and Margolis, 2015; Kallupi et al., 2017; Le Merrer et al., 2009; Lutz and Kieffer, 2013; Toll et al., 2016). MOR activation promotes reinforcement and rewards-seeking, whereas NOPR signaling generally reduces motivational drive under drug exposure or stress conditions (Cippitelli et al., 2020; Kallupi et al., 2017; Koob and Volkow, 2016; Schröder et al., 2014; Wu et al., 2025). Although cell-specific expression patterns of MOR and NOPR have not been fully characterized, current evidence supports their distinct and often opposing actions on rewards and affective processing.

### Scope of this review

The mesocorticolimbic system is composed of heterogeneous neuronal subtypes with distinct molecular, anatomical, and functional properties. Within this circuit, opioid receptors are differentially expressed across excitatory and inhibitory neurons, as well as across projection-defined and genetically distinct cell types (Cole et al., 2024; Driscoll et al., 2020; Erbs et al., 2015; Le Merrer et al., 2009). This cellular diversity contributes to the varied behavioral outcomes observed following selective activation of opioid receptor subtypes.

This review will focus on MOR and NOPR, given their intertwined roles and opposing effects within mesocorticolimbic circuits relevant to opioid use disorder (OUD). This review aims to synthesize recent advances on the cell type–specific organization and functions of MOR and NOPR within the mesocorticolimbic system, emphasizing their differential actions across the VTA, NAc, and PFC. We also highlight how interactions between MOR and NOPR may contribute to maladaptive opioid-use behaviors, and how dual pharmacological targeting of these systems may deliver safer treatments for chronic pain, OUD, and other diseases with motivational symptoms.

## Molecular mechanisms of MOR and NOPR signaling

Mu-opioid receptor and NOPR share approximately 60% sequence homology and couple primarily with G_*i/o*_ proteins (Meunier et al., 1995; Mollereau et al., 1994; Toll et al., 2016). Upon activation by their endogenous ligands (i.e., β-endorphin/enkephalin for MOR and N/OFQ for NOPR), liberated Gα subunits inhibit adenylyl cyclase to reduce intracellular cyclic adenosine monophosphate (cAMP) levels (Fricker et al., 2020; Reeves et al., 2022; Williams et al., 2013). cAMP is a required cofactor for the activity of various cyclic nucleotide-gated channels, guanine exchange factors, and Protein Kinase A. Activation of MOR or NOPR therefore decreases the activity of these channels and enzymes to alter intracellular signaling. Notably, opioid receptor activation decreases phosphorylation of Protein Kinase A substrates such as the cAMP response element-binding (CREB) protein, a transcription factor central to long-term opioid-induced neuroadaptations (Carlezon et al., 2005; Nestler, 2013, 2001).

In parallel, liberated Gβγ subunits rapidly modulate ion channels, including activation of G protein–gated inwardly rectifying potassium (GIRK) channels and suppression of voltage-gated calcium channels. The inhibition of presynaptic N- and P/Q-type voltage-gated calcium channels is one mechanism through which opioid receptors reduce neurotransmitter release (Ikeda, 1996; Wickman and Clapham, 1995; Williams et al., 2013). In addition to these and other G protein-dependent processes, MOR and NOPR can recruit β-arrestins, leading to activation of various kinase signaling pathways. Following GPCR phosphorylation by G protein-coupled receptor kinases (GRKs), β-arrestins can recruit endocytic machinery to promote receptor internalization. MOR’s β-arrestin–mediated signaling is well-established and contributes to tolerance (Bohn et al., 2003; Groer et al., 2011; Mafi et al., 2020; Williams et al., 2013). NOPR also undergoes agonist-induced internalization enhanced by β-arrestin 2 overexpression (Spampinato et al., 2001), and ligand-bias studies further support important functions of NOPR/β-arrestin signaling (Corbani et al., 2004; Malfacini et al., 2015; Spampinato et al., 2002, 2001).

**FIGURE 1:**
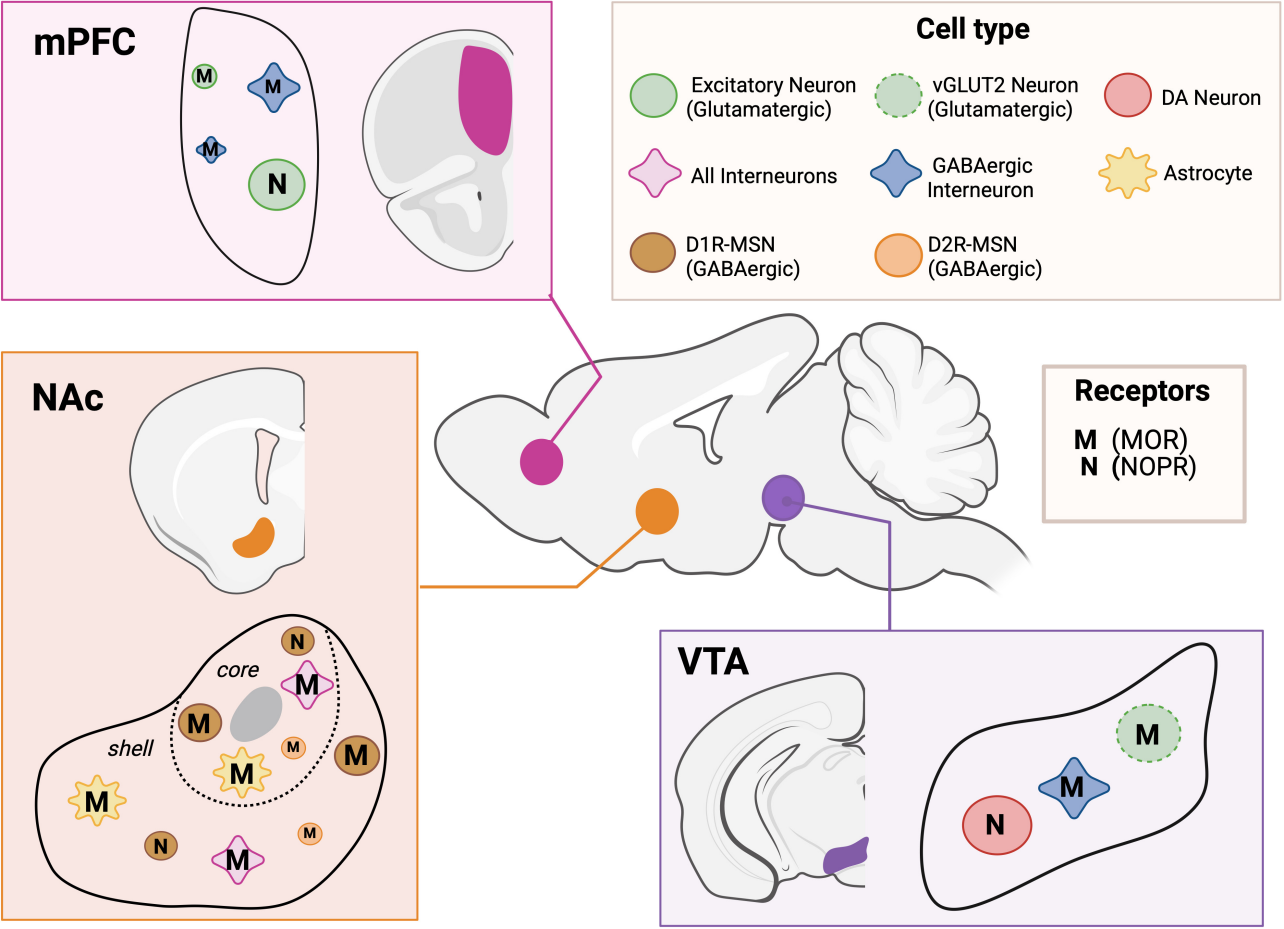
Schematic showing known receptor expression in specific cell type of PFC, NAc, and VTA brain region. DA, dopamine; M (MOR), mu-opioid receptor; D1R-MSN, dopamine D1 receptor; D2R-MSN, dopamine D2 receptor; MSN, medium spiny neuron; N (NOPR), Nociceptin Opioid Receptor; NAc, nucleus accumbens; PFC, prefrontal cortex; VTA, ventral tegmental area; vGLUT2, vesicular glutamate transporter 2. Created with BioRender.com.

Despite their shared G_*i/o*_-coupled signaling, MOR and NOPR display functional divergence attributed to distinct (i) cellular distributions, (ii) subcellular localizations, (iii) ligand availabilities, and (iv) downstream effector coupling. Both receptors are widely expressed throughout the brain, particularly in brain regions controlling motivation, affect, and rewards, such as the cortex, VTA, and NAc (see Figure 1 and Table 1; Darcq and Kieffer, 2018; Ozawa et al., 2015; Witkin et al., 2014). Genetic and pharmacological studies demonstrate that MOR activation is necessary and sufficient for opioid reinforcing effects, as shown by the absence of morphine rewards in MOR knockout mice (Matthes et al., 1996). Conversely, NOPR activation generally suppresses rewards-related processes during stress exposure, negative affect, or opioid withdrawal (Kallupi et al., 2017; Rorick-Kehn et al., 2016). This functional divergence sets the foundation for the following sections, which detail how these receptors operate within the VTA, NAc, and PFC to shape motivation and affective behavior.

**TABLE 1:** Integrated overview of MOR and NOPR cell type-specific expression and functional roles in mesocorticolimbic circuits.

Region	Receptor	Cell type-specific expression	Cellular effects	Behavioral effects	References
VTA	MOR	•GABAergic interneurons (posterior VTA) •vGLUT2-expressing neurons (anterior VTA)	•Decreased GABA •Increased DA	MOR ⇑ •CPP •Increased operant responding MOR ⇓ •Decreased self-administration •Dysphoria, aversion	Gysling and Wang, 1983; Oades and Halliday, 1987; Johnson and North, 1992; Garzón and Pickel, 2001; Margolis et al., 2003; Fields and Margolis, 2015; Svingos et al., 2001a; Bals-Kubik et al., 1993; Devine and Wise, 1994; Nader and van der Kooy, 1997; Bozarth and Wise, 1981
	NOPR	•DAergic neurons •Non-DAergic neurons	•Decreased DA	NOPR ⇑ •Reduced morphine CPP •Reduced motivation for sucrose rewards	Murphy and Maidment, 1999; Lutfy et al., 2001; Maidment et al., 2002; Norton et al., 2002; Zheng et al., 2002; Parker et al., 2019
NAc	MOR	•D1R-MSNs > > D2R-MSNs •GABAergic and ChAT interneurons •Astrocytes	•Decreased glutamate •Decreased GABA	MOR ⇑ •Increased palatable food consumption •Increased social behavior MOR ⇓ •Decreased morphine-induced locomotor sensitization and CPP (D1R-MSNs) •Enhanced opioid-induced sensitization and drug-seeking behavior (D2R-MSNs) •Reduced acquisition of cocaine CPP (D2R-MSNs) •Increased drug-seeking behavior (ChAT interneurons)	Chen et al., 2021; Svingos et al., 1996, 1997; Oude Ophuis et al., 2014; Cui et al., 2014; Won et al., 2023; Yuan et al., 1992; Martin et al., 1997; Hjelmstad and Fields, 2001; Chieng and Williams, 1998; Severino et al., 2020; Remmers et al., 2025; Cornish et al., 2005; Bakshi and Kelley, 1993; Zhang and Kelley, 1997; Katsuura and Taha, 2010; Shin et al., 2010; Trezza et al., 2011; Smith et al., 2018; Resendez and Aragona, 2013
	NOPR	•D1R-MSNs	•Decreased psychostimulant- or opioid-evoked DA release	NOPR ⇑ •Reduced locomotor activity •Reduced operant responding for natural rewards •Increased palatable food intake	Olianas et al., 2008; Murphy et al., 1996; Di Giannuario et al., 1999; Di Giannuario and Pieretti, 2000; Lutfy et al., 2001; Vazquez-DeRose et al., 2013; Narayanan et al., 2004; Wilson et al., 2024; Stratford et al., 1997
PFC	MOR	•Inhibitory neurons > > excitatory neurons	•Decreased GABA •Increased DA •Increased glutamate	MOR ⇑ •Increased locomotor stimulation •Hyperphagia •Increased food-related impulsivity and motivation MOR ⇓ •Reduced morphine-induced CPP •Reduced non-opioid analgesic-induced CPP	Smith et al., 2019; Cole et al., 2024, 2025; Ochandarena et al., 2023; Birdsong et al., 2019; Tejeda et al., 2013; Yang et al., 2020; Mena et al., 2011, 2013; Selleck et al., 2015, 2018; Navratilova et al., 2015; Gomtsian et al., 2018
	NOPR	•Glutamatergic neurons •SST interneurons	•Decreased 5HT and NE	NOPR ⇑ •Increased non-rapid eye movement sleep	Siniscalchi et al., 2002; Sbrenna et al., 1999; Mela et al., 2004; Smith et al., 2019; Morairty et al., 2023

ChAT, cholinergic interneurons; CPP, conditioned place preference; DA, dopamine; MOR, mu-opioid receptor; D1R-MSN, dopamine D1 receptor; D2R-MSN, dopamine D2 receptor; MSN, medium spiny neuron; NOPR, Nociceptin Opioid Receptor; NAc, nucleus accumbens; NE, norepinephrine; PFC, prefrontal cortex; SST, Somatostatin; VTA, ventral tegmental area; vGLUT2, vesicular glutamate transporter 2; 5HT, serotonine; > >, preferentially expressed; ⇑, increase; ⇓, decrease.

## MOR and NOPR expression and regulation of VTA

### Overview of VTA cellular architecture

The VTA is a heterogeneous structure composed of dopamine neurons (55%–65%), GABA neurons (30%–35%), and a smaller population of glutamate neurons (5%) (Morales and Margolis, 2017; Yamaguchi et al., 2007). Some of these neuronal subpopulations respond differently to rewarding or aversive stimuli (Dobi et al., 2010; Lammel et al., 2014; Matsumoto and Hikosaka, 2009; Morales and Margolis, 2017; Nair-Roberts et al., 2008; Pignatelli and Bonci, 2015; Tan et al., 2012). As a central hub of the mesocorticolimbic rewards system, the VTA integrates excitatory and inhibitory inputs to regulate dopamine neuron activity and, consequently, motivation and reinforcement (for a review, see Cooper et al., 2017; Fields and Margolis, 2015; Jalabert et al., 2011; Johnson and North, 1992; Lammel et al., 2014; Thompson et al., 2021).

### MOR expression and function within the VTA

Mu-opioid receptor expression in the VTA was first recognized in the early 1980s, when both intravenous infusion and direct site microinjection of MOR agonists were shown to increase spontaneous activity of dopamine neurons (Gysling and Wang, 1983). Electrophysiological studies later demonstrated that MOR is primarily expressed on VTA GABAergic interneurons such that activation inhibits GABA release onto dopamine neurons. This disinhibition increases dopamine neuron firing and promotes dopamine release in downstream target regions (Fields and Margolis, 2015; Garzón and Pickel, 2001; Gysling and Wang, 1983; Johnson and North, 1992; Oades and Halliday, 1987). The preferential expression of MOR in VTA GABA neurons has also been shown with *in situ* hybridization revealing the presence of *Oprm1* in VTA neurons expressing *Slc32a1* (vesicular GABA transporter) but not *Th* (tyrosine hydroxylase) *Slc6a3* (dopamine transporter) (Galaj et al., 2020). Importantly, however, some studies have found MOR localization in dopamine neurons that project to the PFC, suggesting direct inhibition of specific VTA dopamine neuron subpopulations (Svingos et al., 2001a). Nonetheless, these findings indicate that MOR actions in the VTA are cell type-specific and operate primarily through GABAergic disinhibition.

Anatomical and circuit-level studies showed that long-range GABAergic inputs onto VTA dopamine neurons are differentially sensitive to MOR activation. Inputs from the rostromedial tegmental nucleus are especially sensitive to MOR agonists, undergoing suppression of nearly 50% upon MOR activation (Matsui et al., 2014). By contrast GABA transmission from the NAc and from local interneurons is suppressed by only 18% and 11%, respectively. Other recent studies have found that GABA inputs from the lateral hypothalamus to the paranigral VTA are attenuated by MOR activation (Wu et al., 2025). Taken together, these findings suggest that MOR activation biases VTA dopamine cell activity toward rewards-related projections by suppressing anti-rewards afferents, providing a mechanistic explanation for the robust reinforcing effects observed following intra-VTA MOR stimulation.

### VTA MOR promotes rewards and reinforcement

Local microinjection of MOR agonists, such as morphine or DAMGO, into the VTA of rats reliably produces conditioned place preference (CPP) (Bals-Kubik et al., 1993; Devine and Wise, 1994; Nader and van der Kooy, 1997) and operant responding (Bozarth and Wise, 1981; Zangen et al., 2002). Conversely local MOR antagonism with naloxone prevents opioid reinforcement and self-administration (Bozarth and Wise, 1983, 1981; Britt and Wise, 1983; Ettenberg et al., 1982). These findings demonstrate that MOR signaling in the VTA is both necessary and sufficient for the acute rewarding effects of opioids.

Opioid disinhibition in the VTA leads to increased dopamine levels in the NAc (Devine and Wise, 1994; Spanagel et al., 1992), a common effect of all drugs of abuse (Chiara and Imperato, 1988; Spanagel and Weiss, 1999). Elevated dopamine release from VTA neurons contributes to opioid rewards. Shippenberg et al. (1993) described that morphine CPP is prevented by the lesion of dopamine terminals in the NAc, but not the dorsal striatum, suggesting an essential function for dopamine arising from the VTA. Furthermore, blockade of D1-like but not D2-like receptors in the NAc similarly prevented morphine CPP. Later studies by other groups replicated some but not all these key findings. For example, Nader and van der Kooy (1997) found that dopamine is necessary for CPP to intra-VTA morphine only in animals that were previously made dependent on opioids. Others have shown that CPP to a single exposure of morphine is blocked by intra-NAc infusion of antagonists for either D1-like or D2-like receptors (Fenu et al., 2006). Thus, the opioid exposure history and/or dose are critical in regulating dopaminergic mechanisms of opioid rewards. Consistent with this idea, mice lacking dopamine synthesis displayed reduced CPP to a low dose of morphine (2.5 mg/kg, *s.c.*) but retained CPP at higher doses (5–10 mg/kg) (Hnasko et al., 2005). Notwithstanding potential compensatory changes in tyrosine hydroxylase*-*knockout mice, these findings suggest that opioid rewards and reinforcement involve both dopamine-dependent and dopamine-independent mechanisms. Indeed, when introduced after intravenous heroin self-administration is established, dopamine lesions of the NAc do not abolish reinforced behavior (Pettit et al., 1984), and systemic administration of a dopamine receptor antagonist attenuates but does not abolish heroin self-administration (Ettenberg et al., 1982). Another level of complexity arises from the finding that opioids do not uniformly activate the entire VTA dopamine population but instead recruit specific dopamine neuron subcircuits (Morison et al., 2025). This may further contribute to variability in dopaminergic involvement across behavioral paradigms or laboratories. Taken together, opioid rewards and reinforcement is mediated by both dopaminergic and non-dopaminergic mechanisms, likely involving direct modulation of GABAergic and opioid peptide signaling within mesolimbic circuits.

Chronic opioid exposure induces compensatory adaptations within VTA inhibitory circuits (Bonci and Williams, 1997; Madhavan et al., 2010a,b). Prolonged MOR signaling leads to a homeostatic increase in GABAergic transmission onto dopamine neurons, reducing dopaminergic excitability and blunting rewards responses in the absence of opioids (Madhavan et al., 2010a,b). Opioid withdrawal or MOR blockade increases GABA transmission to suppress dopamine neuron activity, promoting anhedonia, anxiety-like behavior, and conditioned place aversion (Laviolette et al., 2004; Taylor et al., 2016; Ting-A-Kee et al., 2013). Increased activity of VTA GABA neurons during withdrawal shifts the valence from rewarding to aversive, therefore driving avoidance and dysphoria (Laviolette et al., 2004; Ting-A-Kee et al., 2013). These processes are accompanied by long-lasting synaptic adaptations, including reduced inhibitory plasticity, changes in excitatory synaptic composition, and neuroimmune modulation of GABA transmission (Russo and Nestler, 2013; Taylor et al., 2016). Together, these findings demonstrate that VTA MOR signaling encodes both rewarding and aversive dimensions of OUD.

In addition to suppressing GABA transmission onto VTA dopamine neurons, morphine reduces GABA_B_ tone on presynaptic glutamate inputs (Chen et al., 2015). This action leads to potentiated excitatory glutamatergic inputs onto VTA dopamine neurons GABA_B_ receptors (Chen et al., 2015) [although note that some excitatory inputs are also inhibited by MOR (Margolis et al., 2005)]. Consistent with a requirement of local glutamate transmission for opioid rewards, intra-VTA infusion of AMPA and NMDA receptor antagonists prevents morphine-induced CPP (Harris et al., 2004). MOR modulation of GABA and glutamate transmission also regulates somato-dendritic dopamine release in the VTA (Chefer et al., 2009).

Mu-opioid receptor is also expressed in subsets of vesicular glutamate transporter 2 (vGluT2)-expressing neurons within the anterior VTA (McGovern et al., 2023). Activation of these VTA vGluT2 neurons drives place preference and self-stimulation behavior independently of dopamine co-release (Zell et al., 2020). VTA vGluT2 neurons also promote opioids’ reinforcing properties, as chemogenetic inhibition of VTA vGluT2 neurons modulates oxycodone self-administration and cue-induced reinstatement. In addition to acute effects on opioid-seeking, chronic oxycodone exposure produces long-lasting changes in synaptic transmission onto VTA vGluT2 cells (McGovern et al., 2023). Collectively, these data reveal that MOR-dependent signaling in the VTA orchestrates opioid reinforcement and dependence by shaping the excitation-inhibition balance across multiple neuronal populations. MOR-mediated regulation of glutamatergic and non-dopaminergic pathways, alongside GABAergic disinhibition of VTA dopamine neurons, provides a multilayered mechanism by which opioids drive reinforcement, withdrawal-associated aversion and long-term dependence.

### NOPR expression and function within the VTA

Early pharmacological work first showed that N/OFQ signaling inhibits mesolimbic dopamine transmission. Using a dual-probe microdialysis, (Murphy and Maidment, 1999) found that local N/OFQ infusion into the VTA markedly reduced NAc dopamine levels, suggesting direct inhibitory action on VTA dopamine neurons. Consistently, the same group showed that intracerebroventricular administration of N/OFQ decreased NAc dopamine levels and attenuated morphine-induced CPP (Lutfy et al., 2001).

Anatomical evidence supports a local role of NOPR signaling in the VTA. *In situ* hybridization studies reported high NOPR mRNA expression in VTA dopamine neurons (*Th*) (Maidment et al., 2002; Norton et al., 2002). By contrast, N/OFQ is primarily expressed in GABAergic cells, with 10% of *Pnoc* co-localization with *Th* compared to 50%–60% co-expression with GABA synthesizing enzymes (*Gad1/2*) (Norton et al., 2002). These findings suggest that N/OFQ is mainly released by VTA GABA cells to inhibit dopamine neurons. Notably, this system shows plasticity in a dopamine depletion model of Parkinson’s disease. Following 6-OHDA lesions, *Pnoc* levels in the VTA are reduced by ∼80%, accompanied by a compensatory ∼60% increase in *Pnoc* expression (Norton et al., 2002). These findings may have some specificity to the type or extent of lesion, however, as a study using the neurotoxin MPTP found no change in *Pnoc* expression despite a 50% reduction of VTA dopamine neurons (Gouty et al., 2010). Interestingly, *Pnoc* knockout mice display reduced MPTP-induced dopamine neuron loss in the substantia nigra pars compacta (Arcuri et al., 2018; Brown et al., 2006), and pharmacological inhibition of NOPR confers similar protective effects in PD models (Arcuri et al., 2018; Marti et al., 2004; Mercatelli et al., 2019).

Complementing these anatomical observations, electrophysiological studies indicate that N/OFQ directly inhibits VTA neurons. In midbrain slices, NOPR agonists reduce firing and hyperpolarize both dopaminergic and non-dopaminergic neurons (Zheng et al., 2002). The inhibitory actions of NOPR agonists are concentration-dependent, insensitive to tetrodotoxin and naloxone, and blocked by NOPR antagonism, confirming that they arise from NOPR rather than classical opioid receptors. In addition, N/OFQ decreases measures of GABA release probability onto VTA dopamine neurons, raising the possibility of coincidental disinhibition, at least in some circuits (Zheng et al., 2002). Together, these early findings support a model in which N/OFQ, released from local GABAergic interneurons, stimulates post-synaptic NOPR expressed on dopamine neurons of the VTA to inhibit mesolimbic output (Mercatelli et al., 2019).

Recent work has extended this model to show that N/OFQ inhibition of VTA neurons depends on their projection targets. N/OFQ application generated outward currents (putatively mediated by GIRK channels) in PFC- and NAc-projecting dopamine neurons. By contrast, posterior anterior cingulate cortex (ACC)-projecting neurons displayed small inward currents, suggesting excitatory responses in a distinct subpopulation of cells. These findings reveal that NOPR signaling does not uniformly inhibit VTA output but exerts projection-dependent modulation of dopamine neuron excitability (Driscoll et al., 2020). These findings refined earlier models derived from microdialysis studies (Lutfy et al., 2001; Murphy and Maidment, 1999), confirming that the inhibitory influence of the NOPR system on dopamine release originates within the VTA itself.

### VTA NOPR attenuates rewards and reinforcement

Recent work from the Bruchas lab has used cell type-specific and circuit-resolved approaches to clarify how N/OFQ signaling regulates VTA function and motivational behavior. Using *Pnoc*-Cre genetically engineered mouse lines, they traced a discrete population of *Pnoc*-expressing neurons within the paranigral VTA that project directly onto neighboring dopamine neurons. Optogenetic activation of paranigral VTA *Pnoc* neurons reduced operant responding for sucrose under a progressive ratio schedule, suggesting that endogenous N/OFQ release locally suppresses dopaminergic activity to limit rewards drive (Parker et al., 2019). These findings provide the first cell type–specific evidence that the VTA N/OFQ system reduces motivation, consistent with the inhibitory effects on dopamine observed in early pharmacological studies (Lutfy et al., 2001; Murphy and Maidment, 1999). The development of a genetically encoded fluorescent sensor for nociceptin (NOPLight) now enables real-time monitoring of endogenous N/OFQ release in freely moving animals. Using fiber photometry, chemogenetic activation of paranigral VTA *Pnoc* neurons increases NOPLight fluorescence. This effect is abolished by a NOPR antagonist, confirming specific actions of the endogenous ligand on the receptor-based biosensor. Further studies revealed that endogenous nociceptin dynamics are bidirectional: NOPLight signal decreases during sucrose consumption and increases during tail-lift stress. These data demonstrate state-dependent modulation of N/OFQ release within paranigral VTA rather than uniform activation during rewards (Zhou et al., 2024). Together, these studies describe circuits through which VTA N/OFQ signaling reduces motivation for natural rewards, setting the foundation for targeted manipulations in models of OUD.

## MOR and NOPR expression and regulation of NAc

### Overview of NAc cellular architecture

The nucleus accumbens (NAc), the primary target of mesolimbic dopamine projections, is generally required for the behavioral effects arising from VTA modulation. The role of opioid receptors in the NAc in controlling rewards-related behaviors is well-established (Goeders et al., 1984; Olds, 1982, p. 198; Van Der Kooy et al., 1982). The NAc is subdivided into the “core” and “shell” regions (Burke et al., 2017; Haber, 2011) and is composed of ∼95% GABAergic medium spiny neurons (MSNs; also referred as “spiny projection neurons”). MSNs are largely segregated into two subtypes depending on dopamine receptor expression and canonical projection targets (Gerfen et al., 1990). “Direct pathway” MSNs express D1 receptors (D1R-MSN) and project to the VTA. In parallel, “indirect pathway” MSNs express D2 receptors (D2R-MSN) and project to the ventral pallidum (VP) (Pardo-Garcia et al., 2019; Robertson and Jian, 1995). Importantly, this anatomical distinction is nuanced, as a substantial proportion of D1R-MSNs also project to the VP. Although activation of D1R-MSNs is generally associated with promoting motivation and rewards, and D2R-MSNs with behavioral inhibition, recent evidence indicates some overlapping functions and context-dependent roles for both MSN subtypes (Flanigan and LeClair, 2017; Kupchik et al., 2015; Salery et al., 2020; Zachry et al., 2024). The remaining neuronal population (<5%) is composed of several types of interneurons, including fast-spiking interneurons, low-threshold spiking interneurons, and cholinergic (ChAT) interneurons. These interneurons exert strong inhibitory and modulatory control over MSNs, shaping both spontaneous and evoked network activity (Abudukeyoumu et al., 2019; Berke, 2011; Burke et al., 2017; Faust et al., 2015; Tepper et al., 2010, 2004).

In addition to interneuron-mediated inhibition, MSNs themselves participate in local microcircuit regulation by forming functional collateral synapses with one another (Taverna et al., 2004). Studies report an asymmetry in connectivity probability between MSN subtypes, whereby D2R-MSNs exhibit a higher likelihood of forming collateral synapses than D1R-MSNs (Planert et al., 2010; Taverna et al., 2008). D2R-MSNs provide lateral inhibitory inputs onto neighboring D1R-MSNs, contributing to competitive regulation of striatal output (Dobbs et al., 2016; Gallo et al., 2018; Lalchandani et al., 2013). Moreover, MSN subtypes differentially express endogenous opioid peptides, with D1R-MSNs primarily releasing dynorphin (KOR ligand) and D2R-MSNs releasing enkephalin (MOR/DOR ligand) (Besson et al., 1990; Creed et al., 2016; Gerfen et al., 1990; Soares-Cunha et al., 2020). These peptides are locally released and engage opioid receptors within the NAc microcircuit, providing an intrinsic source of opioid modulation that shapes MSN activity and network dynamics.

### MOR expression and function within the NAc

Autoradiographic*, in situ* hybridization, immunocytochemical, and multi-omics studies (Arvidsson et al., n.d.; Charbogne et al., 2017; Chen et al., 2021; Delfs et al., 1994; Mansour et al., 1995a,b; McLean et al., 1986; Svingos et al., 1996; Tempel and Zukin, 1987; Zastawny et al., 1994) show robust MOR expression throughout the NAc. Early work reported modestly higher binding and *Oprm1* levels in the shell compared to the core (Delfs et al., 1994). More recent spatial transcriptomic analyses reveal *Oprm1* enrichment in the ventromedial NAc with a graded anterior-posterior increase (Chen et al., 2021). At the ultrastructural level, MOR is localized in extrasynaptic sites and on presynaptic GABA terminals contacting MSNs (Svingos et al., 1996, 1997). MOR is expressed preferentially in D1R-MSNs, but also in D2R-MSNs and subsets of interneurons (Chen et al., 2021; Cui et al., 2014; Oude Ophuis et al., 2014; Svingos et al., 2001b). Recent studies using a MOR-mCherry knock-in mouse further revealed MOR expression in over 60% of astrocytes in the NAc and other brain structures, as well as in a significant fraction of GABAergic parvalbumin (PV)-expressing interneurons (Won et al., 2023).

Ultrastructural evidence supports the complexity of endogenous opioid signaling within the NAc. Enkephalin-immunoreactive boutons originating from local MSNs are more abundant in the shell than in the core (Meredith et al., 1993). Enkephalin terminals primarily form asymmetric synapses onto MSN spines, but extrasynaptic and axo-axonal contacts also exist. More recently, snRNA-seq and RNAscope analyses identified a transcriptionally distinct cluster of MSNs marked by expression of the carbohydrate-sulfotransferase gene *Chst9* (Andraka et al., 2024), suggesting additional heterogeneity within MOR-expressing MSNs that may confer specific roles in opioid signaling in the NAc.

Mu-opioid receptor stimulation in the NAc decreases excitatory transmission, although the specific glutamatergic afferent are not resolved (Hjelmstad and Fields, 2001; Martin et al., 1997; Yuan et al., 1992). In the dorsomedial striatum, MOR reduces glutamate release at optogenetically isolated inputs from the mPFC, the ACC, and the basolateral amygdala (Muñoz et al., 2020), suggesting that MOR may similarly regulate multiple presynaptic glutamate inputs in the NAc. Postsynaptic MOR decreases calcium channel function (Chieng and Bekkers, 2001). The MOR agonist DAMGO also enhances NMDA receptor currents (Martin et al., 1997), although this effect is not fully blocked by the MOR antagonist CTOP (Martin et al., 1997), suggesting potential involvement of additional opioid receptors. In addition, MOR activation also decreases GABA release (Chieng and Williams, 1998; Yuan et al., 1992), an effect that is independent of cAMP signaling and is potentiated by opioid dependence and withdrawal. At the microcircuit level, neuropeptide Y-expressing interneurons are highly sensitive to MOR activation (Retzlaff and Rothwell, 2022). MOR activation suppresses neuropeptide Y interneuron firing and GABA release, with a greater effect at synapses onto D1R-MSNs. These inhibitory effects are more pronounced in females, raising the possibility that sex-dependent differences in MOR modulation of inhibitory interneurons may contribute to sex differences observed in opioid-related behaviors.

Mu-opioid receptors in the NAc display rapid trafficking and signaling adaptations following opioid exposure. Acute morphine increases intracellular MOR localization in dendritic compartments without modifying surface receptor density, suggesting early subcellular redistribution (Haberstock-Debic et al., 2003). Systemic morphine administration increases ERK phosphorylation in the NAc shell in mice (Rosas et al., 2016; Valjent et al., 2004) [albeit, with less consistent effects in rats (Xu et al., 2012)]. Intra-NAc shell infusion of the GluN2B antagonist ifenprodil or the MEK inhibitor U0126 blocks both ERK phosphorylation and the expression of morphine CPP, therefore identifying the GluN2B-ERK pathway in D1R-MSN as a critical mechanism for long-term synaptic plasticity and opioid rewards learning (Xu et al., 2012).

Chronic morphine administration induces persistent adaptations in NAc glutamate transmission, including long-term changes in NMDA currents and presynaptic glutamate release onto MSNs. Morphine causes significant cell type- and pathway-specific glutamatergic plasticity in the NAc (Graziane et al., 2016). Morphine exposure generates silent synapses in D2R-MSNs by internalizing AMPA receptors from synapses which are preferentially eliminated during withdrawal. This process results in a NAc output bias toward rewards seeking and reinforcement mediated by D1R-MSNs and decreased excitatory drive onto D2R-MSNs (Graziane et al., 2016). At the circuit level, a pathway from the paraventricular thalamus to the NAc medial shell is both necessary and sufficient for withdrawal-induced aversion. Through insertion of GluA2-lacking AMPA receptors, morphine selectively potentiates thalamic input onto D2R-MSNs (Zhu et al., 2016). Optogenetic depotentiation of this pathway abolishes withdrawal symptoms. Consistent with this finding, GluA2-lacking AMPA receptors in the NAc shell are required for negative-affective but not somatic behavioral effects of morphine withdrawal (Russell et al., 2016). On the other hand, repeated morphine exposure enhances glutamatergic transmission and GluA2-lacking AMPA receptor expression in D1R-MSNs. Reversing these adaptations prevents reinstatement (Hearing et al., 2016). D2R-MSNs show increased intrinsic excitability despite decreased synaptic input following repeated morphine injections, maintaining firing outputs through homeostatic compensation (McDevitt et al., 2019). These cell type-specific adaptations evolve across drug exposure stages in the NAc shell: acute morphine enhances AMPA receptor-mediated transmission in D1R-MSNs, spontaneous withdrawal increases AMPA receptor signaling in D2R-MSNs, and re-exposure after abstinence triggers rapid endocytosis of GluA2-containing AMPA receptors in D1R-MSNs (Madayag et al., 2019). Overall, D2R-MSN-centered adaptations driving withdrawal-related aversion and D1R-MSN potentiation supporting rewards and relapse.

### Behavioral roles of MOR signaling within the nucleus accumbens

Pharmacological and genetic studies demonstrate that NAc MOR is critical for behavioral effects of opioids (Fields and Margolis, 2015; Self and Stein, 1992; Zhang and Kelley, 2000). Morphine-evoked increases in striatal dopamine, as well as locomotor sensitization and CPP are abolished in MOR knockout mice and rescued by selective MOR overexpression in NAc D1R-MSNs (Cui et al., 2014). These effects are likely due to expression of MOR in MSN axons in the VTA or elsewhere, as MOR agonist infusion into the NAc itself is not reinforcing (Zangen et al., 2002). Nonetheless, MOR deletion in D1R-MSNs decreases morphine- and oxycodone-induced hyperlocomotion and sensitization, without affecting drug-seeking (Severino et al., 2020). MOR expression in D1R-MSNs is therefore necessary and sufficient for the stimulant and rewarding properties of opioids, whereas drug-seeking involves additional sets of receptors or neuronal populations. By contrast, MOR knockout in D2R-MSNs enhances opioid-induced sensitization and drug-seeking (Severino et al., 2020) but does not affect rewards learning (Remmers et al., 2025). Notably, deletion of MOR from D2R-MSNs attenuates acquisition of cocaine CPP (Remmers et al., 2025), indicating drug-specific roles for NAc MOR signaling. Deletion of MOR from ChAT interneurons does not affect hyperlocomotion but does increase drug-seeking behavior following opioid self-administration (Severino et al., 2020), supporting cell type-specific functions of MOR signaling within the NAc to shape opioid-related behaviors.

Importantly, MSN subtypes themselves constitute an intrinsic source of opioid signaling within the NAc, with dynorphin released by D1R-MSNs and enkephalin by D2R-MSNs (Besson et al., 1990; Creed et al., 2016; Gerfen et al., 1990; Soares-Cunha et al., 2020). Local release of these endogenous opioid peptides can modulate striatal microcircuit activity through engagement of opioid receptors expressed within the NAc (Al-Hasani et al., 2015; Banghart et al., 2015; Nam et al., 2019; Tejeda and Bonci, 2019). This endogenous opioid tone likely interacts with MOR signaling to shape MSN activity and may contribute to the cell type–specific behavioral effects observed following MOR manipulation.

Subregional MOR distribution further shapes opioid self-administration. MORs within the caudal NAc contribute more to heroin reinforcement than those in the rostral region (Martin et al., 2002). Sex differences also influence NAc MOR functions. Lesions of NAc MOR impair acquisition and reinforcement of heroin self-administration in males, whereas extended-access heroin intake is selectively decreased in females (Bossert et al., 2023), suggesting sex differences in MOR signaling for acquisition versus maintenance of opioid use. NAc MOR signaling also contributes to polysubstance abuse. Inhibition of NAc MOR reduces self-administration of a heroin-cocaine combination (Cornish et al., 2005) and combined low doses of MOR and D1R antagonists suppress intake, highlighting that MOR-dopamine interactions shape polydrug reinforcement.

Beyond drug reinforcement, MOR signaling in the NAc regulates natural rewards processing. Local MOR stimulation increases the palatability and consumption of preferred foods (Bakshi and Kelley, 1993; Katsuura and Taha, 2010; Shin et al., 2010; Zhang and Kelley, 1997). Subregion-specific effects, termed hedonic “hotspots” and “coldspots,” have been described and reviewed in detail elsewhere (Castro and Berridge, 2014; Castro and Bruchas, 2019; Peciña and Berridge, 2005). Activation of NAc MORs also enhances affiliative social behaviors (Smith et al., 2018; Trezza et al., 2011) and pair-bond formation (Resendez and Aragona, 2013). Conversely, blockade of NAc MOR prevents social behaviors (Smith et al., 2018; Toddes et al., 2024; Trezza et al., 2011). Collectively, these results show that MORs in the NAc play a central role in modulating the rewarding and reinforcing properties of both natural rewards and drugs of abuse.

### NOPR expression and function within the NAc

Within the NAc, mRNA expression and NOPR-stimulated GTPγS assays both indicate that NOPR is preferentially expressed in the medial shell subregion (Anton et al., 1996; Berthele et al., 2003; Mollereau et al., 1994; Neal et al., 1999a,b; Slowe et al., 2001). Pharmacological and biochemical evidence indicates that NOPR is present both on presynaptic terminals arising from the VTA and within D1R-MSNs (Olianas et al., 2008). In isolated striatal synaptosomes, N/OFQ inhibits dopamine synthesis and phosphorylation of tyrosine hydroxylase through a pathway dependent on Protein Kinase A. In addition, N/OFQ inhibits postsynaptic responses driven by D1R activation in NAc slices and cultures, including accumulation of cAMP, as well as downstream phosphorylation of NMDA receptor and CREB. By contrast, N/OFQ does not affect cAMP signaling stimulated by A2A adenosine receptors, which are expressed selectively in NAc D2R MSNs. Together, these findings suggest that NOPR signaling preferentially dampens D1R-MSN activity, providing a mechanism by which NOPR attenuates rewards and reinforcement.

Concerning N/OFQ, a recent study using *Pnoc*-tdTomato mice indicates that cells that express N/OFQ at any point during development cluster in the NAc core and dorsal medial shell (Hueske et al., 2024). Approximately 60%–70% of striatal *Pnoc* cells co-express *Drd1*, with minimal overlap with markers of D2R-expressing neurons. Notably, ∼25% of NAc *Pnoc* cells express neither *Drd1* nor *Drd2*, suggesting relative enrichment in interneurons. An important caveat to these findings is that only 1%–5% of tdTomato-positive cells expressed *Pnoc* in adulthood, suggesting that the vast majority of labeling in this study occurred during development. The limited expression of *Pnoc* in the NAc in adulthood raises the possibility that NAc N/OFQ may also arise from unidentified extraneous sources.

Studies on the ability of NOPR activation to suppress dopamine release in the NAc yield contradictory results. While some *in vivo* microdialysis studies show decreases in dopamine overflow (Koizumi et al., 2004; Lutfy et al., 2001; Murphy et al., 1996), others report minimal or no effect (Di Giannuario et al., 1999; Di Giannuario and Pieretti, 2000; Vazquez-DeRose et al., 2013). A recent *ex vivo* fast scan cyclic voltammetry study found no effect of N/OFQ on evoked dopamine release in NAc slices (Driscoll et al., 2020). Despite mixed findings regarding basal dopamine release, consensus has been found regarding the ability of NOPR activation to reduce psychostimulant- or opioid-evoked dopamine increase in the NAc (Murphy et al., 1996; Di Giannuario et al., 1999; Di Giannuario and Pieretti, 2000; Lutfy et al., 2001; Vazquez-DeRose et al., 2013; Narayanan et al., 2004). To date, whether N/OFQ modulates glutamate or GABA transmission within the NAc has not been reported.

### Behavioral effects of NOPR signaling within the nucleus accumbens

Relatively few studies have assessed the behavioral actions of NOPR within the NAc. Infusion of N/OFQ into the NAc reduces locomotor activity, albeit to a lesser extent than infusions into the VTA (Narayanan et al., 2004). Consistent with systemic or intracerebroventricular administration, N/OFQ infusion into the NAc shell suppresses drug-induced dopamine release (Vazquez-DeRose et al., 2013). N/OFQ in the NAc also modulates motivation for natural rewards. Notably, N/OFQ infusion into the NAc core reduces lever-pressing for sugar in effort-based choice tasks (Wilson et al., 2024). Interestingly the same manipulation increases passive intake of palatable food, and others have shown that microinjection of N/OFQ into the NAc shell also increases food intake (Stratford et al., 1997). Together, these findings suggest N/OFQ differentially regulates food-motivated behavior depending on motivational context. In addition, mice with an anxious phenotype display greater NOPR-induced GTPγS incorporation in the NAc (Le Maître et al., 2006), raising the possibility that NAc NOPR might mediate changes in rewards-seeking in negative affective states.

## MOR and NOPR expression and regulation of PFC

### Overview of cellular architecture of medial PFC

The rodent medial PFC (mPFC) is classically segmented into three compartments: the ACC, the prelimbic cortex, and the infralimbic cortex. These cortical regions serve as key motivational and affective hubs, integrating intracortical processing with extensive connections to mesolimbic brain regions. Changes in synaptic proteins in PFC regions have been linked to many psychiatric diseases, particularly substance use disorders. Prolonged drug exposure causes drug-induced plasticity in the PFC (Van den Oever et al., 2010), leading to synaptic and circuit-wide modifications that promote addiction-related behaviors. Recent studies are beginning to define the cellular and molecular mechanisms through which endogenous and exogenous opioids modify PFC function.

### MOR expression and function within the PFC

Mu-opioid receptor is expressed by both inhibitory and excitatory cell populations in neocortex (Smith et al., 2019). MOR expression in excitatory neurons appears to display a gradient across the rostro-caudal axis, such that MOR expression within the PFC is much more localized to inhibitory neurons (Cole et al., 2025; Ochandarena et al., 2023; Smith et al., 2019). Consequently, MOR activation generally suppresses GABAergic signaling in the PFC, disinhibiting cortical circuitry (for a review see Cole et al., 2024). MOR is also expressed on excitatory afferents arriving in the mPFC, through which it can directly regulate synaptic transmission onto pyramidal cells. Studies in PFC slices have shown that MOR agonists can reverse increases in glutamate transmission triggered by nicotine (Lambe et al., 2003) and serotonin agonists (Béïque et al., 2007; Marek and Aghajanian, 1998). Serotonin-induced increases in glutamate transmission in the PFC are reduced by lesions of the mediodorsal nuclei of the thalamus (Marek et al., 2001); therefore, it seems likely that MOR in presynaptic thalamic terminals reduces excitatory drive in the PFC. Indeed, PFC MOR binding is reduced following lesions of the thalamus (Marek et al., 2001; Sahin et al., 1992), suggesting that thalamic terminals contain a high proportion of PFC MOR. Recent studies employing optogenetics have also found evidence for high expression of MOR within thalamocortical synapses. DAMGO reduces thalamic-evoked excitatory postsynaptic currents onto pyramidal cells along with thalamic feedforward inhibition (Birdsong et al., 2019, p. 2; Jaeckel et al., 2024). The endogenous peptide agonist met-enkephalin exerts similar effects across the thalamocortical circuit (Hervert and Birdsong, 2023). Within the PFC, endogenous expression of enkephalin is nearly exclusive to inhibitory neurons, particularly those that express vasoactive intestinal peptide (Smith et al., 2019), but it is also possible that the PFC receives extraneous sources of enkephalin.

At the subcellular level, PFC MOR signals through somatodendritic and presynaptic compartments to regulate cell excitability and neurotransmitter release. Interneurons that express somatostatin are the most sensitive to MOR agonists, displaying outward currents mediated by potassium channel activation along with reductions in GABA release onto pyramidal cells (Cole et al., 2025). Recent findings suggest that PFC MOR exhibits cell type-specific differences in subcellular localization. Perhaps the strongest example of this divergent pre- and postsynaptic signaling occurs in fast-spiking PV interneurons. Using *ex vivo* slice electrophysiology, several labs have shown that MOR activation fails to suppress GABA release from PV-interneurons in cortical areas. In the prelimbic cortex, ACC, and sensory and motor cortices, MOR activation does not inhibit PV-mediated optically-evoked IPSCs (Birdsong et al., 2019; Caccavano et al., 2025; Cole et al., 2025), despite producing hyperpolarizing outward currents in these cells. This dissociation appears to be region-specific, as DAMGO suppresses transmission from PV interneurons in the orbitofrontal cortex (Lau et al., 2020) and areas of the hippocampus (Caccavano et al., 2025; He et al., 2021). Considering the evidence that presynaptic opioid receptors are more resistant to desensitization than postsynaptic receptors (Coutens and Ingram, 2023; Pennock et al., 2012), these findings may imply that SST cells and PV cells will display major differences in response to chronic opioid use. In addition, a high proportion of cholecystokinin interneurons express MOR; yet, functional studies show only modest effects of MOR activation relative to other interneuron subtypes (Cole et al., 2025). This finding motivates additional studies to examine how PFC MOR may affect other aspects of cell physiology, intracellular signaling, and gene expression. Local administration of MOR agonists also increases dopamine overflow in the mPFC (Tejeda et al., 2013). While the mechanism and requisite receptor populations have not been determined, this effect on dopamine likely occurs through disinhibition of VTA dopamine cell activity. It is therefore possible that dopamine contributes to long-term changes to the PFC function following chronic opioid exposure.

### Cortical MOR signaling following opioid exposure

Chronic opioid exposure leads to changes in PFC MOR function. Chronic exposure to MOR agonists often leads to decreased sensitivity of MORs upon subsequent activation. Long-term exposure to heroin decreases DAMGO-stimulated GTPγS binding in the thalamus and amygdala but not in the PFC (Maher et al., 2005), suggesting that presynaptic MOR on excitatory inputs to PFC may be most sensitive to tolerance. Indeed, recent studies using optogenetics revealed that inputs from medial thalamic nuclei to the ACC display attenuated MOR function following chronic morphine exposure via osmotic minipump (Jaeckel et al., 2024). Chronic morphine decreased MOR’s ability to suppress thalamic glutamate transmission onto both pyramidal cells and interneurons, with some sex differences in the magnitude of the effect. MOR tolerance was not observed in genetically engineered mice with a phosphorylation-deficient receptor. These findings indicate that morphine dependence confers sex-specific changes in MOR signaling in thalamocortical synapses, with potential ramifications for pain, affective behaviors, and opioid-seeking.

Several studies have observed strengthened glutamate transmission at rewards-related PFC projection targets following exposure to MOR agonists. In slices, *ex vivo* morphine treatment can promote glutamate release from mPFC pyramidal cells to dopaminergic VTA neurons, indicated by an increase in the amplitude of excitatory postsynaptic currents and a corresponding decrease in paired-pulse ratio (Yang et al., 2020). The potentiation of glutamate transmission appears to be related to the expression of GABA_B_ receptors on mPFC terminals in the VTA. By contrast, nearby terminals arising from the lateral hypothalamus do not express GABA_B_ and are not potentiated by morphine. These findings suggest that disinhibitory MOR signaling within VTA can differentially alter the weight of long-range excitatory inputs. Furthermore, inhibition of these mPFC projection neurons reduces morphine-induced locomotor stimulation and CPP, supporting a role in acute rewarding properties of opioids.

A similar phenomenon has been observed in extinguished rats with a history of heroin exposure. An acute challenge with heroin increases excitatory synaptic strength from PFC to NAc, measured by *in vivo* excitatory post-synaptic field potentials (Shen et al., 2011). This potentiation of PFC-evoked activity in the NAc is accompanied by alterations in dendritic spine morphology and recruitment of GluN2B-containing NMDA receptors. Blockade of GluN2B-NMDA receptors prevents heroin-induced changes to excitatory synaptic remodeling as well as reinstatement of heroin seeking. Consistent with an upregulation of glutamate signaling following chronic MOR activation, western blot analysis of postmortem brain tissue from men suffering from opioid and polysubstance use disorders showed a significant increase in the level of GluN1 and GluN2A subunits in the mPFC (Daneshparvar et al., 2019). Thus, there are several mechanisms by which MOR activation increases glutamatergic transmission in mPFC circuits to mediate opioid seeking and relapse behaviors.

Dysregulation of PFC MOR has also been observed in humans with a history of opioid use. For example, postmortem tissue samples from men who use heroin exhibit dysregulated *Oprm1* splice variant mRNAs compared with control subjects (Brown et al., 2022). In parallel, the authors demonstrate similar splice variant dysregulation in male rats that underwent 10 days of heroin self-administration. The finding in rats indicates that this phenomenon is conserved across species and provides evidence that the change occurs in response to opioid exposure rather than as a pre-existing vulnerability trait. One category of splice variants that showed a large change was the full-length C-terminal variants, the function of which has been implicated in altered morphine-induced tolerance, physical dependence, rewards behavior, and locomotor activity (Xu et al., 2017).

### mPFC MOR signaling regulates state-dependent appetitive motivation and feeding

A substantial body of work demonstrates that MOR signaling within the rodent mPFC regulates motivation for natural rewards [reviewed in Baldo (2016)]. A series of pharmacological studies from the Baldo lab showed that direct infusion of DAMGO into the mPFC robustly increases food intake. In male rats, DAMGO infusion into the ventromedial PFC (vmPFC) induces locomotor stimulation and augments carbohydrate consumption compared to palatable fat-enriched diets, even in sated animals (Mena et al., 2011). This finding is not reproduced by stimulation of DOR or KOR agonists and is specific to the vmPFC, as DAMGO infusions into lateral orbital or anterior motor cortex do not affect feeding. In a subsequent study, (Mena et al., 2013) used cFos expression following intra-vmPFC MOR stimulation to begin to characterize the circuitry involved in MOR-mediated stimulation of feeding behavior. Intra-vmPFC DAMGO infusions increase cFos expression in the dorsomedial tuberal hypothalamus, enhancing the activity of hypocretin/orexin-containing neurons. vmPFC MOR-induced feeding behavior was inhibited by blockade of NMDA receptors in the lateral perifornical hypothalamic areas, while blockade of AMPA receptors in the NAc shell potentiated vmPFC MOR-induced feeding and motivation for sucrose. Together, these findings indicate that vmPFC MOR-induced feeding involves dissociable roles of glutamate signaling within the hypothalamus and ventral striatum.

Subsequent work expanded on the role of cortical MOR signaling in appetitive behavior, implicating vmPFC MOR in state-dependent control of feeding behavior. Selleck et al. (2015) used two sucrose-reinforced tasks: differential reinforcement of low response rates and progressive ratio to test the ability to suppress impulsive-like responses and motivational value of the rewards, respectively. Shifting rats from 2-h food deprivation (“low drive state”) to 18-h food deprivation (“high drive state”) increases impulsive responding in the task. In male rats, this increased impulsivity is attenuated by blockade of vmPFC opioid receptors with the non-specific antagonist methylnaloxonium. This finding indicates vmPFC opioid receptors are necessary for diminished inhibitory control that occurs in a food-deprived high-drive state. In contrast, stimulation of MORs in the vmPFC in male rats in the low drive state increases impulsivity and motivation, features characteristic of the high drive state. Interestingly, monoamine stimulation by amphetamine did not affect either task, consistent with earlier findings that intra-vmPFC dopamine blockade does not alter food intake (Mena et al., 2011). While direct activation of the vmPFC dopamine system does not recapitulate the effects of MOR stimulation, the Baldo lab has shown that MOR and dopamine systems in the vmPFC interact to mediate food-related behavior. Intra-vmPFC administration of the D1R antagonist SCH23390 reverses the DAMGO-induced increase in food motivation but not intake (Selleck et al., 2018). These findings demonstrate that D1 receptor signaling is required for the increase in motivation driven by vmPFC-dependent MOR.

### Cortical MOR contributions to the affective and motivational dimensions of pain relief

Mu-opioid receptor signaling within the ACC is critical in regulating the affective and motivational components of pain. Several studies have established that endogenous opioid signaling in the ACC is necessary for the rewarding effects of pain relief, positioning cortical MORs as key mediators of negative reinforcement rather than primary nociceptive processing (Gomtsian et al., 2018; Navratilova et al., 2015, 2024). A study by Navratilova et al. (2024) demonstrates this dissociation by showing that microinjection of naloxone into the rostral ACC prevents CPP to non-opioid pain relief while not limiting the reversal of mechanical allodynia. Similarly, selective ablation of MOR-containing neurons in the rostral ACC prevents rewarding properties of pain relief while maintaining reversal of thermal and tactile hypersensitivity. Importantly, lesions of rostral ACC MOR neurons do not affect the formation of cocaine CPP, confirming specificity for rewarding effects of pain relief. In a subsequent study, the authors did not observe effects of ACC MOR activation on tactile, thermal, or mechanical stimulation in uninjured or neuropathic rats (Gomtsian et al., 2018); yet, ACC MOR activation elicits CPP in injured rats nonetheless. Overall, these findings suggest that rostral regions of the ACC selectively modulate affective qualities of pain without affecting evoked responses.

### NOPR expression and function in the PFC

Nociceptin/orphanin FQ receptor is widely expressed throughout the cortex (Yao et al., 2023). In human brain tissue, high levels of [^3^H]-N/OFQ binding and *Oprl1* expression are observed in the PFC and cingulate cortex (Berthele et al., 2003; Peluso et al., 1998). High NOPR expression in mPFC is also corroborated by positron emission tomography studies in humans (Khan et al., 2018) and autoradiography in rodents (Florin et al., 2000). Single-cell transcriptomic analysis of the mouse motor and visual cortices reveals widespread expression of *Oprl1* across many glutamatergic cell types along with some somatostatin-expressing GABAergic cells (Smith et al., 2019). Therefore, NOPR expression in the mPFC contrasts with MOR due to primary expression in excitatory neurons, although both NOPR and MOR are expressed at high levels in somatostatin-expressing cells. Functional studies of mPFC NOPR have largely focused on monoamine release. NOPR activation decreases serotonin and norepinephrine release from PFC synaptosomes to a greater extent than MOR or other opioid receptors (Mela et al., 2004; Sbrenna et al., 1999; Siniscalchi et al., 2002). To our knowledge, whether NOPR regulates excitatory or inhibitory synaptic transmission in the PFC has not yet been reported.

### Emerging evidence for cortical NOPR involvement in aversive and withdrawal-related states

Much less is known about the involvement of mPFC NOPR in OUD and other substance use disorders. Rodent studies have hinted at a link between cortical N/OFQ signaling and alcohol use disorder (Ploj et al., 2000), though changes to N/OFQ and NOPR levels have not been corroborated by human postmortem studies (Kuzmin et al., 2009; Lutz et al., 2015). Available evidence points toward a modulatory role in the aversive and affective components of opioid addiction and withdrawal. In rats subcutaneously implanted with two 75 mg morphine pellets, conditioned naloxone-precipitated morphine withdrawal decreased N/OFQ expression in the frontal cortex (Walker et al., 2002). By contrast, increased N/OFQ expression occurred following mild unconditioned opioid withdrawal, which consisted of 2 days of subcutaneous 5 mg/kg morphine injections followed by naloxone. While conditioned and unconditioned opioid withdrawal produced opposing adaptations to PFC N/OFQ expression, it is unclear whether these changes arise from differences in the route or length of morphine exposure. To separate stress-related effects from opioid withdrawal, separate rats were exposed to unpredictable electric footshock. The authors observed a trending, yet insignificant, elevation in N/OFQ from repeated electric footshock, suggesting that the adaptations to N/OFQ expression are not related to a general stress response. Interestingly, adult rats exposed to chronic social defeat stress show decreased expression of *Pnoc* and *Oprl1* transcripts in the PFC (Pizzagalli et al., 2025), suggesting that changes to N/OFQ signaling may depend on the stress modality, intensity, or duration. Recent evidence from non-human primates supports this role for cortical N/OFQ signaling in behavioral responses to aversive stimuli. In squirrel monkeys that underwent Pavlovian fear conditioning, the NOPR agonist SCH-221510 attenuates conditioned stimulus-induced neuronal activation in PFC-amygdala circuitry and increased functional connectivity among PFC subregions (Hur et al., 2025). Collectively, these data indicate a potential role for mPFC N/OFQ in regulating negative affect and/or OUD-associated stress reactivity.

Nociceptin/orphanin FQ receptor signaling in the cortex may also be subject to circadian regulation. NOPR activation promotes non-rapid eye movement sleep and increases EEG slow-wave activity, implicating this receptor in sleep-wake control (Morairty et al., 2023). Interestingly, the same study referenced prior findings showing that circadian patterns of gene expression in the human prefrontal cortex change with aging (Chen et al., 2016), suggesting that NOPR-mediated effects in the PFC may vary according to circadian phase or sleep state. Such time-dependent modulation could influence the receptor’s role in affective regulation and stress reactivity, processes that are themselves under strong circadian control.

## Interplay between MOR and NOPR signaling

Spatially and functionally distinct distributions of MOR and NOPR within the rewards circuit enable the integration of disinhibitory and inhibitory signaling processes, establishing a dynamic balance between facilitation and inhibition of rewards-related behavior. Rather than acting as independent systems, MOR and NOPR signaling appear to form an integrated regulatory axis controlling motivational circuit excitability. This interplay provides a mechanistic basis for their contrasting behavioral outcomes and highlights the therapeutic potential of coordinated targeting of MOR and NOPR signaling to preserve analgesia while limiting addictive liability.

### Functional opposition and circuit-level integration

Mu-opioid receptor and NOPR signaling often counterbalance one another, producing opposing outcomes on rewards, reinforcement, and analgesia. MOR usually promotes rewards, analgesia, and reinforcement, whereas NOPR acts as a homeostatic brake on motivational circuits (Cippitelli et al., 2020; Devine et al., 1996; Kallupi et al., 2017; Koob and Volkow, 2016; Parker et al., 2019; Zhou et al., 2024). Although close associations between MOR and NOPR have been reported in dorsal root ganglia (Majumdar et al., 2011; Ozawa et al., 2015; Pan et al., 2002; Toll et al., 2016), there is no evidence for such receptor complexes within the mesocorticolimbic circuit. Instead, their opposing behavioral outcomes likely arise from a combination of distinct cellular expression patterns and differential intracellular signaling mechanisms.

In the VTA, MOR expression is enriched in GABA interneurons and in some glutamatergic inputs to dopamine cells, resulting in opioid-induced disinhibition of dopamine neurons (Margolis et al., 2003). Conversely, NOPR is expressed by dopamine neurons, enabling a N/OFQ to directly reduce dopamine neuron firing (Parker et al., 2019). This opposition also applies to the PFC, where preferential expression of MOR in GABA interneurons disinhibits PFC activity (Birdsong et al., 2019; Cole et al., 2025), while high expression NOPR in glutamatergic pyramidal cells dampens excitatory transmission (Florin et al., 2000; Peluso et al., 1998; Siniscalchi et al., 1999).

Importantly, functional opposition between MOR and NOPR does not require strict anatomical segregation or independent circuit engagement. When co-expressed within the same cell, both receptors couple to G_i/o_ signaling pathways and may compete for shared intracellular effectors, including G proteins, βγ subunits, and downstream kinase signaling cascades (Connor and Christie, 1999; Toll et al., 2016). Differences in receptor density, ligand availability, desensitization kinetics, or coupling efficiency could therefore bias signaling dominance within the same neuron or microcircuit. Studies in heterologous systems demonstrate that sustained NOPR activation can lead to heterologous desensitization of MOR through mechanisms dependent on G protein receptor kinases and Protein Kinase C (Mandyam et al., 2003, 2002). Although comparable mechanisms have not yet been demonstrated in mesocorticolimbic neurons, these findings provide a plausible cellular framework through which NOPR activation may constrain MOR-driven excitation and plasticity. Together, these observations suggest that MOR–NOPR opposition emerges not only from circuit architecture but also from how shared inhibitory signaling mechanisms are deployed at the synaptic level.

Beyond rewards circuitry, NOPR activation modulates spinal and supraspinal nociceptive processing. Thus, NOPR-targeted interventions may influence analgesia in parallel with motivational processes, although these effects are at least partially dissociable from mesocorticolimbic mechanisms (Toll et al., 2016).

### Translational implications: dual MOR-NOPR targeting and bifunctional ligands

The complementary expression patterns and functional opposition of MOR and NOPR support a systems-level framework in which engagement of both receptors enables fine-tuning of rewards processing, motivation, and analgesia. Selective MOR agonists such as morphine and fentanyl provide potent analgesia but carry high risks of tolerance, dependence, and respiratory depression. In contrast, selective NOPR agonists produce anxiolytic effects and attenuate the reinforcing properties of addictive drugs but can also affect motivation for natural rewards (Parker et al., 2019; Sukhtankar et al., 2014; Varty et al., 2005; Zaveri, 2016). Complementary behavioral pharmacology has motivated the design of compounds that engage both receptors, aiming to preserve analgesia while reducing MOR-driven addictive potential. Indeed, bifunctional MOR/NOPR agonists such as cebranopadol, AT-121, and BU08028 show strong antinociception with a more favorable side-effect profile than classical MOR agonists (Dahan et al., 2017; de Guglielmo et al., 2017; Ding et al., 2016; Khroyan et al., 2011; Linz et al., 2014; Tzschentke et al., 2019). As highlighted by Parker and Bruchas (2018), the most compelling motivation for bifunctional MOR/NOPR agonists is not simply additive analgesia, but the prospect of dissociating opioid analgesia from the physiological and motivational liabilities driven by MOR alone.

#### Cebranopadol

Cebranopadol is a high-efficacy MOR agonist and partial NOPR agonist that produces long-lasting analgesia in rodent models of acute and neuropathic pain (Christoph et al., 2017). Cebranopadol has clinical efficacy with a favorable tolerability profile in patients with different pain modalities (Göhler et al., 2019; Tzschentke et al., 2019); however, preclinical work reveals that the rewarding properties of cebranopadol are not eliminated by its NOPR activity. Cebranopadol induces CPP in rodents (de Guglielmo et al., 2017), indicating rewards learning. Furthermore, in drug-discrimination studies in rats, cebranopadol generalizes to morphine and this effect is blocked by naloxone (Tzschentke and Rutten, 2018), suggesting that cebranopadol can exert some subjective effects of MOR agonists. Consistent findings were obtained in a clinical study in humans. Recreational opioid users found that low-to-moderate doses of cebranopadol (200–400 μg) produced drug-liking ratings similar to placebo, while higher doses (800 μg) produced effects comparable to hydromorphone (8 mg, but not 16 mg) (Göhler et al., 2019). These data suggest that cebranopadol has reduced, but not absent, abuse potential relative to classical MOR agonists. Importantly, recent preclinical evidence demonstrates that cebranopadol markedly attenuates heroin self-administration and stress-induced reinstatement of heroin seeking, while exhibiting lower reinforcing efficacy than heroin across fixed- and progressive-ratio schedules, supporting its potential utility in opioid use disorder (Cannella et al., 2024). Moreover, its respiratory depressant effects are shorter-lived than its analgesic actions (Dahan et al., 2017), consistent with a somewhat improved safety profile compared with pure MOR agonists rather than absence of risk. Taken together, cebranopadol reduces but does not eliminate abuse potential, and improves but does not nullify respiratory risk.

#### BU08028

BU08028, a buprenorphine-derived ligand, displays MOR partial and NOPR efficacious agonism. In mice, BU08028 is a very potent and long-lasting analgesic but increases locomotor activity and produces significant CPP, indicating strong MOR-mediated rewarding effects despite its NOPR component (Khroyan et al., 2011). In contrast, in non-human primates it produces robust antinociception with markedly reduced respiratory depression, cardiovascular side effects, reinforcing strength, and physical dependence compared with classical MOR agonists (Ding et al., 2016). A recent focused review (Wang et al., 2022) synthesizes these findings, emphasizing BU08028’s dual MOR/NOPR engagement as the mechanistic basis for reduced respiratory suppression, low abuse liability, and sustained antinociception. The review also highlights preliminary evidence that BU08028 can blunt rewards-like behavior induced by opioids and psychostimulants, and may even suppress ethanol-driven effects, supporting potential utility beyond analgesia.

#### BPR1M97

A recently developed dual MOR/NOPR full agonist, BPR1M97 behaves as a MOR full agonist and a G protein–biased NOPR agonist in cell-based assays (Chao et al., 2020). BPR1M97 shows potent antinociception across multiple rodent pain assays with reduced respiratory, cardiovascular, and gastrointestinal side effects relative to morphine. BPR1M97 also produces lower CPP, reduced locomotor activation, and milder naloxone-precipitated withdrawal. These findings suggest that ligand-specific NOPR bias may contribute to improved tolerability.

#### AT-121

AT-121 is a balanced MOR/NOPR partial agonist. In rhesus monkeys, AT-121 produces morphine-level antinociception without respiratory depression, physical dependence, or reinforcing effects in self-administration paradigms (Ding et al., 2018). AT-121 also suppresses oxycodone self-administration. While published studies are more limited than other bifunctional ligands, AT-121 currently offers the strongest primate evidence for analgesia without reinforcement or withdrawal signs. Importantly, however, some structurally related analogs like AT-201 produce only modest effects on heroin reinstatement and reacquisition (Bossert et al., 2024). Differences in G protein signaling efficacy and receptor phosphorylation have been identified as potential mechanisms underlying key behavioral effects (Dasgupta et al., 2022). This highlights the importance of behavioral domain, sex, pharmacokinetics, and dosing window, and cautions against assuming uniform benefit of MOR-NOPR bifunctional ligands.

#### BU10038

This compound derived from a naltrexone analog (now called PPL-138) was first reported as a mixed MOR/NOPR partial agonist based on the GTPγS binding assay (Kiguchi et al., 2019). Like the functional profile of BU08028 and AT-121, BU10038 lacks reinforcing effects and the risk of respiratory depression in primates. Interestingly, recent rodent studies have found that BU10038 is effective in reducing cocaine intake and alcohol self-administration (Cippitelli et al., 2025, 2022), indicating great potential as a novel medication for substance use disorder.

## Future directions and conclusion

Beyond their analgesic potential, MOR-NOPR interactions have emerged as a promising target for modulating motivational and affective dimensions of OUD. While accumulating evidence indicates that NOPR activation can dampen drug seeking, stress-induced relapse, and negative affect, major gaps remain in our understanding of how MOR and NOPR signaling are organized across cell types, circuits, and behavioral states.

A key unresolved question concerns circuit specificity. Recent work has identified discrete neuronal ensembles within mesolimbic circuits that differentially encode drug versus natural rewards (Tan et al., 2024), yet the respective contributions of MOR and NOPR signaling within these populations remain unknown. Determining whether distinct receptor mechanisms govern opioid reinforcement versus natural rewards processing, and how these mechanisms shift across craving, withdrawal, and relapse states, represents a critical next step. In addition, the role of MOR and NOPR signaling in prefrontal circuits governing impulse control and relapse vulnerability remains poorly defined but may help bridge molecular mechanisms with higher-order behavioral vulnerability in OUD. Finally, translation beyond rodents will require systematic validation in non-human primates to establish whether analgesia–rewards dissociation is conserved across species.

Emerging single-cell, spatial, and circuit-resolved approaches now provide the necessary tools to bridge molecular signaling, neural activity, and behavior. Integrating these methods will be essential for defining how MOR and NOPR signaling converge within defined neuronal populations and for guiding the rational development of safer opioid therapeutics. In conclusion, targeting the MOR-NOPR axis offers a mechanistically grounded path toward analgesics with reduced abuse liability. Progress will depend on moving beyond receptor-level pharmacology toward a circuit- and cell type-specific understanding of opioid action.
